# Both the middle and distal sections of the urethra may be regarded as optimal targets for ‘outside-in’ transobturator tape placement

**DOI:** 10.1007/s00345-014-1261-1

**Published:** 2014-02-17

**Authors:** Michał Bogusiewicz, Marta Monist, Krzysztof Gałczyński, Magdalena Woźniak, Andrzej P. Wieczorek, Tomasz Rechberger

**Affiliations:** 1 2nd Department of Gynecology, Medical University of Lublin, Jaczewskiego 8, 20-954 Lublin, Poland; 2 Department of Pediatric Radiology, Medical University of Lublin, Lublin, Poland

**Keywords:** Stress urinary incontinence, Suburethral sling outcome, Tape position, Ultrasound

## Abstract

**Purpose:**

To investigate whether the position of the tape under the urethra may influence ‘outside-in’ transobturator sling (TOT) outcome.

**Methods:**

The study comprised 141 women who underwent TOT for clinically and urodynamically proved stress urinary incontinence. The postoperative ultrasound examination with an endovaginal biplane probe was performed before discharging the patients from hospital. The measurements obtained described the position of the tape relative to the urethra and pubic symphysis, as well as anatomical relationships in the anterior compartment.

**Results:**

Ninety-six (68.1 %) patients were cured, 27 (19.1 %) significantly improved, and in 18 cases (12.7 %), the surgery failed. The tape position under the midurethra (40–70th percentile of the urethral length) or distal urethra (>70th percentile) coincided with better results (cure rate 67.1 and 82.4 %, respectively) than the location in the proximity of the bladder neck (<40th percentile) (21.4 % cured, *p* = 0.0015 and *p* < 0.001, respectively). However, the risk of failure was the lowest when the tape was located under the distal urethra. Other ultrasonographic findings were not related to treatment results.

**Conclusions:**

The highest failure rate for ‘outside-in’ TOT is associated with the location of the tape under the proximal third of the urethra. Both the middle and distal sections of the urethra may be regarded as targets for transobturator tape placement.

## Introduction

Suburethral synthetic slings are nowadays a first-choice method for surgical treatment of female stress urinary incontinence (SUI) [1]. Since the role of the tape is to reinforce weakened pubouretheral ligaments, its insertion under the middle section of the urethra has been recommended [2–4]. Indeed, several studies confirm the advantages of midurethral tape position [5–8]. The part of the urethra termed ‘the high pressure zone,’ which is estimated to lie between 53 and 72 % of the functional urethral length, has been proposed as the optimal target for suburethral tape placement [9, 10]. Conversely, other reports question any relationship between the tape position relative to the urethra and the sling outcome, emphasizing that the gap between the tape and the pubic symphysis plays a more important role [11–15]. Favorable sling results have also been linked to ultrasonographic parameters reflecting tape tension, such as the compression of the urethra by the tape, described as ‘encroachment,’ or a shorter distance between the urethra and the tape [5–7, 13]. However, due to variable ultrasound techniques used and parameters measured in these studies, their findings are not directly comparable, and the impact of the tape position on the sling outcome is still a matter of discussion.

Previous reports demonstrated that a biplane endovaginal high-frequency ultrasound probe can be successfully used for a detailed evaluation of the urethral structures and surrounding tissues, as well as for a precise identification of the tape’s position under the urethra [16–18]. The aim of the present study was to investigate whether the position of the tape under the urethra can influence the results of an ‘outside-in’ transobturator sling (TOT). In addition, our objective was to determine whether the position and outcome of the sling are related to the ultrasonographic parameters representing anatomical relationships in the urethra.

## Materials and method

Between April 2009 and June 2010, 179 women, who had undergone ‘outside-in’ TOT for clinically and urodynamically proved SUI, were prospectively enrolled in the study. Exclusion criteria comprised a previous pelvic reconstructive or anti-incontinence surgery, symptoms of urgency, detrusor overactivity, and anterior vaginal wall prolapse stage III or higher, assessed according to the Pelvic Organ Prolapse Quantification (POP-Q) system [19].

A TOT procedure with a monofilament tape was performed by means of an IVS-04 M device (Covidien^®^) according to the technique described by Delorme [20]. Fifty-eight patients concomitantly underwent posterior vaginal wall colporrhaphy or posterior mesh reinforcement due to prolapse stage II or III.

Before discharging the patients from hospital, two investigators (M.B. and A.P.W.) conducted endovaginal ultrasound with an ultrasound scanner ProFocus 2202 (B–K Medical, Herlev, Denmark) and a biplane transducer (type 8848; B–K Medical, Herlev, Denmark), frequency range 5–12 MHz, by a standardized technique, as described previously [17, 18]. The 3D volumes obtained were analyzed offline with software provided by the producer. The analysis was conducted independently by two investigators (M.B. and A.P.W.) who were blinded to clinical data and to each other’s measurements. The mean value of the two measurements of each parameter was used for analysis. According to the classification proposed by Shoukri and Pause [21], good to excellent inter- and intraobserver agreement was noted. Interclass correlation coefficients ranged from 0.713 to 0.932. Interobserver reliability was assessed for one investigator (M.B.).

**Fig. 1:**
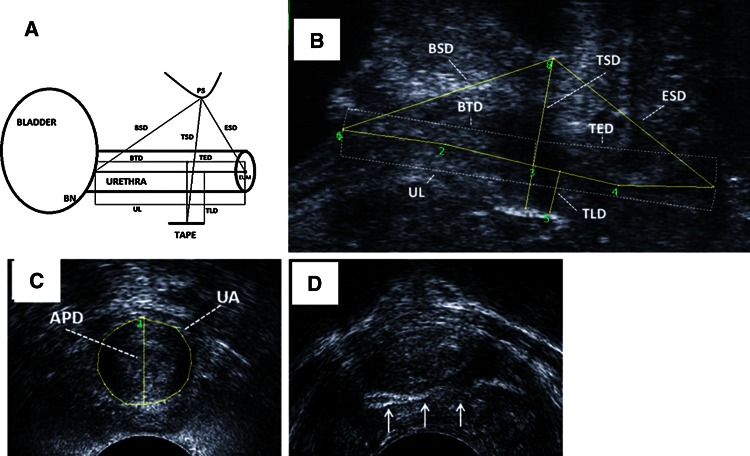
**a**, **b** Measurements taken at the sagittal plane: *UL* urethral length, *BTD* the bladder neck–tape distance, *TED* tape–external urethral meatus distance, *BSD* bladder neck–pubic symphysis distance, *ESD* external urethral meatus–pubic symphysis distance, *TSD* tape–pubic symphysis distance, *TLD* tape–urethral lumen distance, *BN* bladder neck, *PS* pubic symphysis, *EUM* external urethral meatus. **c** Measurements taken at the axial plane: *APD* anterior–posterior diameter, *UA* urethral area. **d** Presence of urethral ‘encroachment’

The following measurements were taken at the sagittal plane (Fig. 1a, b):

1. Urethral length (UL) was measured from the bladder neck to the external meatus, along the urethral longitudinal axis. Setting the reference point at the midpoint of the tape, the bladder neck tape (BTD) and tape external urethral meatus (TED) distances were determined during the measurement of the UL. The position of the tape relative to the percentile of the UL was calculated, assuming the bladder neck as the proximal end of the urethra.
2. Bladder neck–symphysis pubis distance (BSD)—measured from the bladder neck to the lowest margin of the symphysis pubis.
3. External urethral meatus–symphysis pubis distance (ESD)—measured from the external urethral meatus to the lowest margin of the symphysis pubis.
4. Tape–symphysis pubic distance (TSD)—measured from the midpoint on the tape to the lowest margin of the symphysis pubis.
5. Tape–urethral lumen distance (TLD)—measured as the shortest distance between the tape and the urethral lumen.

At the axial plane, the anterior–posterior diameter (APD) and urethral area (UA) were measured at midurethra, and the whole urethra was inspected for the presence of ‘encroachment’ by the tape (Fig. 1c, d).

One hundred and thirty-six women were able to attend, and 5 were readmitted to hospital because of recurrent stress urinary incontinence before the scheduled period of 24 months. Overall, 141 women were included in further analysis.

Assessments of the sling outcome were performed after 24 months, consisting of a cough test with the patient supine and standing with a comfortably full bladder, and a standard 1-h pad test, as described previously [22]. Patients were considered completely cured if they were free of all subjective SUI symptoms, and if the cough tests and the pad test were negative. The operation was considered as a failure if the patient still reported urine leakage during increases in intra-abdominal pressure, or if the cough test or pad test was positive. In the improved group, the cough test was negative, but patients still reported occasional urinary leakage, or the pad test was negative, but the increase in pad weight was greater than zero and smaller than 1 g.

Ethical approval for the study was obtained from the Ethical Committee of Medical University in Lublin (KE-0245/29/2008). All the patients gave their informed consent prior to inclusion in the study.

Statistical analysis was performed with Statistica Statsoft, version 8 package, using the unpaired *t* test, the Mann–Whitney U test, *χ* ^2^, Pearson’s correlation coefficient, or ANCOVA, as appropriate. A *p* < 0.05 was considered statistically significant.

## Results

### Demographic and clinical variables

The analysis of data from 141 patients with known sling outcomes showed that 96 (68.1 %) patients were cured, 27 (19.1 %) significantly improved, and 18 (12.7 %) experienced no significant improvement after the surgery. The patients for whom the surgery was ineffective were older and more frequently postmenopausal than those in the other groups. In addition, women with treatment failure had a higher BMI in comparison with those who were completely dry. Other demographic and clinical factors did not significantly influence sling results (Table 1).

**Table 1:** Demographic, clinical and ultrasonographic parameters in relation to sling results; mean ± SD (range) and *n* (%)

	Cured (*n* = 96)	Improved (*n* = 27)	Failed (*n* = 18)	Statistical significance
Age (years)	56.5 ± 10 (31–78)	58.4 ± 9.2 (42–77)	63.0 ± 10.8 (44–80)	Cured versus failed: *p* = 0.014
BMI (kg/m^2^)	27.4 ± 4.6 (19.3–39.9)	29.7 ± 5.5 (20.4–42.7)	31.3 ± 5.3 (23.4–45.7)	Cured versus failed: *p* = 0.0018 Cured versus improved: *p* = 0.034
Parity	2.5 ± 1.1 (0–8)	2.6 ± 1.1 (1–5)	2.5 ± 0.7 (1–4)	NS
Menopause	57 (59.4 %)	19 (70.4 %)	3 (83.3 %)	Cured versus improved: *p* = 0.006
Previous hysterectomy	5 (5.2 %)	3 (11.1 %)	1 (5.5 %)	NS
ISD (VLPP < 60 ml H_2_O)	4 (4.2 %)	0	2 (11.1 %)	NS
Posterior vaginal prolapse (POPQ ≤ 1)	5 (27.7 %)	11 (40.7)	31 (32.3 %)	NS
De novo urgency	10 (10.4 %)	0	1 (5,6 %)	NS
Acute postoperative urinary retention	5 (5.2 %)	0	1 (5,6 %)	NS
Tape location (percentile of the urethral length)	65.0 ± 13.6	61.2 ± 15.4	46.9 ± 18.0	Cured versus failed *p* < 0.001 improved versus failed *p* = 0.006
Urethral length (UL) (mm)	37.9.0 ± 4.9	37.9 ± 5.8	40.3 ± 4.7	NS
Urethral ‘encroachment’	21 (21.9 %)	2 (7.4 %)	5 (27.8)	NS
Tape–urethral lumen distance (TLD) (mm)	4.9 ± 1.4	5.1 ± 1.6	5.2 ± 1.6	NS
Tape–symphysis pubic distance (TSD) (mm)	19.6 ± 3.6	20.9 ± 4.0	20.8 ± 4.1	NS
Bladder neck–symphysis pubis distance (BSD) (mm)	23.8 ± 3.8	24.0 ± 4.1	21.5 ± 4.6	Cured versus failed *p* = 0.024*
External urethral meatus–symphysis distance (ESD) (mm)	19.7 ± 4.4	20.8 ± 4.4	23.2 ± 6.1	Cured versus failed *p* = 0.017*
Anterior–posterior diameter (APD) (mm)	12.9 ± 1.3	13.0 ± 1.7	12.6 ± 1.0	NS
Urethral area UA (cm^2^)	1.5 ± 0.3	1.5 ± 0.4	1.4 ± 0.2	NS

* Difference were no longer statistically significant after ANCOVA was performed

### Tape position

**Fig. 2:**
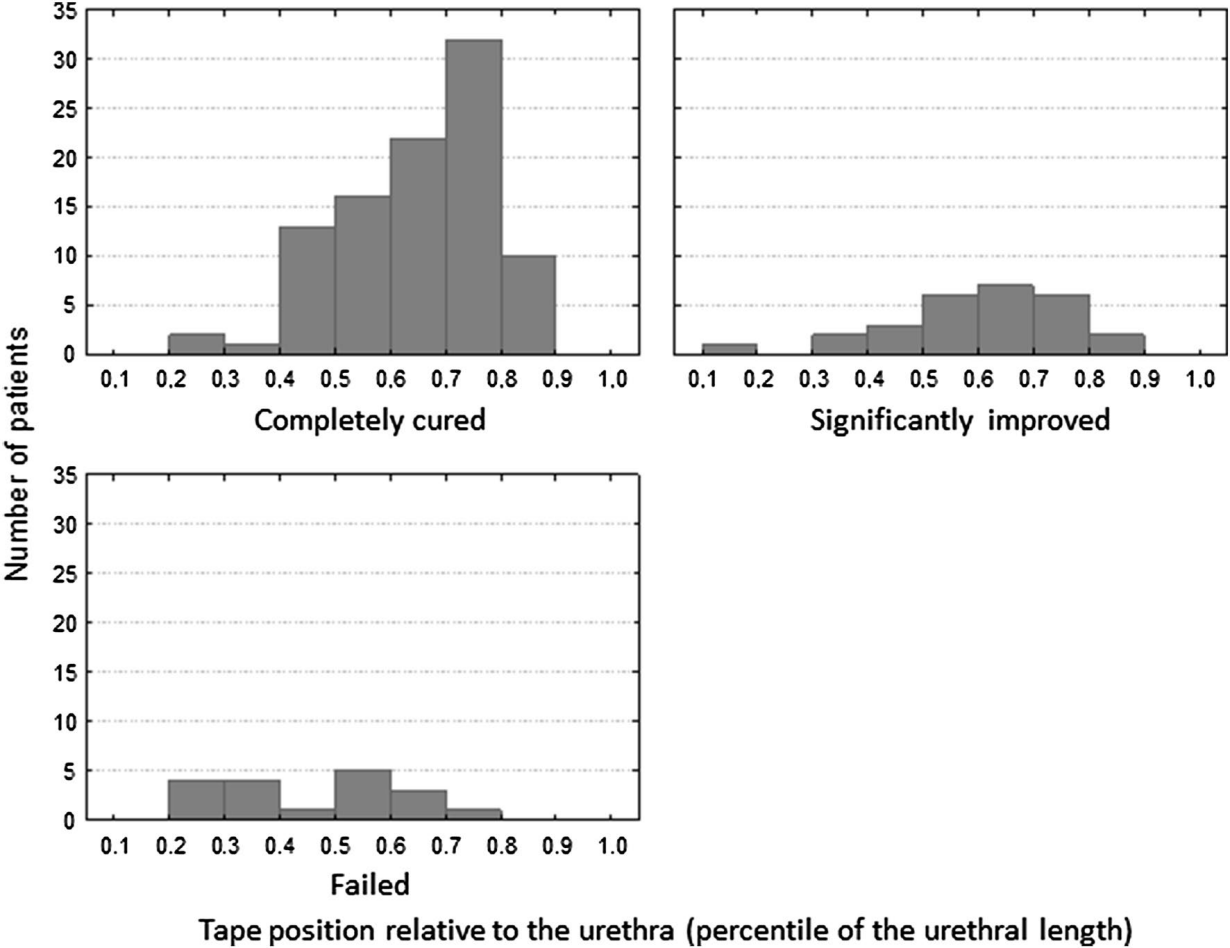
Distribution of tapes under the urethra

The mean tape position was significantly more distal in the cured and improved patients than in those who did not benefit from the surgery (Table 1). The bar charts showing the distribution of the tape’s position relative to the urethral length, presented in Fig. 2, indicate that most of the cured and improved patients had tapes located distally to the 40th percentile. To explore the impact of the location of the tape under the urethra on sling results, the urethra was divided into the proximal (<40th percentile), middle (40th–70th percentile) and distal sections (>70th percentile).

The rate of success, defined as a complete cure, was the highest when the tape was positioned under the distal urethra, but the advantage of this location, compared with the midurethral one, did not reach statistical significance (*χ* ^2^ = 3.62, *p* = 0.057). Both distal and middle positions were superior to the proximal placement. The incidence of treatment failure was the lowest when the tape was implanted under the distal urethra, but the position of the tape under the middle section of the urethra also coincided with a lower failure rate than the position under the proximal section (Table 2). The position of the tape under the urethra was not related to anatomical parameters, such as the urethral length, bladder neck–symphysis pubis distance or external urethral meatus–symphysis pubis distance. The analysis of covariance (ANCOVA) showed that although age (*p* = 0.046) and BMI (*p* = 0.002) influenced treatment results, they had no compounding influence on the effect of the tape position (*p* < 0.001).

**Table 2:** Treatment results in relation to tape position under the urethra; *n* (%)

Treatment result	<40 Percentile of the UL (*n* = 14)	40–70 Percentile of the UL (*n* = 76)	>70 Percentile of the UL (*n* = 51)	Statistical significance
Cured	3 (21.4 %)	51 (67.1 %)	42 (82.4 %)	>70 Percentile versus <40 percentile: *χ* ^2^ = 18.68, *p* < 0.001 40–70 Percentile versus <40 percentile: *χ* ^2^ = 1.02, *p* = 0.0015
Improved	3 (21.4 %)	16 (21.1 %)	9 (17.6 %)	NS
Failed	8 (57.1 %)	9 (11.8 %)	1 (2 %)	>70 Percentile versus <40 percentile: *χ* ^2^ = 27.52, *p* < 0.001 40–70 Percentile versus <40 percentile: *χ* ^2^ = 8.56, *p* = 0.0034 >70 Percentile versus 40–70 percentile: *χ* ^2^ = 7.28, *p* = 0.007

The position of the tape in relation to the urethral lumen (tape–urethral lumen distance) or pubis symphysis (tape–symphysis pubic distance), as well as urethral ‘encroachment’, did not influence sling efficacy. Urethral encroachment coincided with a shorter tape–urethral lumen distance (3.8 ± 0.9 vs. 5.3 ± 1.4 mm, *p* < 0.001) and a shorter tape–symphysis pubic distance (17.7 ± 1.2 vs. 20.8 ± 3.3 mm *p* < 0.001), which indicate that all these parameters reflect the tension applied during tape placement. Among other parameters determined by ultrasound, a longer bladder neck–symphysis pubis distance and a shorter external urethral meatus–symphysis pubis distance were associated with better results. These differences, however, were no longer statistically significant after ANCOVA was performed, using tape position, age, and BMI as compounding factors.

### Complications

In 11 (7.8 %) women, de novo urgency developed after the surgery. None of the ultrasonographic parameters was significantly related to this condition. Only one woman with de novo urgency had a visible tape ‘encroachment’. One patient with the tape located close to the bladder neck was subjected to sling incision. The patients with de novo urgency were older than those free of this complaint (65.6 ± 6.9 vs. 57.0 ± 10.1; *p* = 0.006).

Five patients had acute postoperative urinary retention requiring a prolonged stay in hospital, and one of them underwent tape incision because of voiding difficulties, but remained continent.

## Discussion

The results of our study show a strong relationship between the position of the tape and the outcome of ‘outside-in’ TOT. In line with other reports, we observed that the location of the tape under the proximal urethra, close to the bladder neck, is related to a greater risk of treatment failure [5–8, 23]. In our population, the position of the tape proximally to the 40th percentile of the urethral length was associated with failure in almost 60 % of the patients, whereas the position of the tape distally to the 40th percentile resulted in a substantially higher cure rate. Surprisingly, unlike other investigators, we did not find any association of poorer outcomes with the distal tape position. In fact, the location of the tape at >70th percentile of the urethral length resulted in the lowest failure rate.

It has been observed that the location of the tape close to the bladder neck may predispose to de novo urgency [18, 24]. Statistical analysis of the results of the present study does not support this association. Although patients with the tape under the proximal urethra who develop de novo urgency are encountered in clinical practice, a study comprising a larger population of such patients is needed to prove that the tape position plays a causative role in this complication.

Observations relating sling results to the location of the tape under the urethra imply that greater control over the place where the tape is positioned should produce a higher rate of success. For the retropubic procedure, Kociszewski et al. [10] recommend the preoperative measurement of the urethral length by introital ultrasound and suggest that the site for the beginning of the suburethral incision be calculated according to the one-third rule. These steps are to maximize the probability that TVT is placed under the distal end of the midurethra. The results of the present study indicate that for TOT, it is more important to avoid placing the tape too proximally than to aim at the middle section of the urethra. In most patients, this can be achieved by following the standard rules. However, because of a considerable anatomical variability, including the bony pelvis [25], proximal tape positioning may occur in some patients despite the application of an appropriate surgical technique. To prevent tape displacement during final adjustment, we introduced a modification of the transobturator sling procedure, consisting in the application of 2 additional sutures to the periurethral tissue, 0.5 cm laterally on each side of the midurethra, and between 1.0 and 1.5 cm from the external urethral meatus, which fixate the tape. This step significantly increased the clinical efficacy of the procedure [22].

Reports published previously link the tension of the tape to sling results. The compression of the urethra by the tape at rest and during straining, also termed ‘encroachment,’ associates with better results, but also with a higher incidence of de novo urgency and voiding dysfunctions [5–7, 15, 26]. We did not observe any association of urethral ‘encroachment’ at rest either with surgical results or with voiding difficulties. As in other studies, urethral compression among our patients coincided with a shorter distance between the tape and the urethra, and with a shorter distance between the tape and the pubic symphysis.

Although according to the integral theory, a correction of the vaginal axis may play a major role in the continence status [2]; patients after a posterior vaginal wall repair for prolapse stage II or III were not excluded from the study. This approach was based on the lack of evidence that an isolated repair of the rectocele may affect sling efficacy, as well as on the observation of Yang et al. [26] that anterior colporrhaphy with mesh reinforcement is the only concomitant pelvic surgical procedure that affects the position and motion of the bladder neck or the TOT position. We believe that the study population including such patients is more representative of women encountered in everyday clinical practice.

### Conclusions

The highest failure rate of ‘outside-in’ TOT coincides with the location of the tape under the proximal third of the urethra, whereas both the middle and distal sections of the urethra may be regarded as targets for tape placement during this procedure. The distance of the tape from the pubic symphysis and urethral ‘encroachment’ do not seem to affect TOT results.
